# Autointoxication in Carnivora1Read before the semi-annual meeting of the Pennsylvania State Veterinary Medical Association, Pittsburg, September, 1898.

**Published:** 1898-11

**Authors:** S. J. J. Harger

**Affiliations:** Professor of Veterinary Anatomy and Zoötechnics, University of Pennsylvania


### AUTOINTOXICATION IN CARNIVORA.[1]

By S. J. J. Harger, V.M.D.,
PROFESSOR OF VETERINARY ANATOMY AND ZOÖTECHNICS, UNIVERSITY OF PENNSYLVANIA.

Autointoxication may take place in all the domestic species, but it seems to be most frequent in carnivora. Our knowledge of certain poisonous microbic secretions (*toxins*, *toxalbumins*) which, when introduced into the system, cause certain well-defined diseases, lead us to suggest that, on account of microbic and fermentative action in the digestive tract as well as certain chemic processes in the functions of nutrition, accompanied by the formation of compounds capable of poisoning the tissues, the body is in constant danger of intoxication both from without and within. This thought is most forcibly suggested to me by certain obscure and usually fatal diseases of the house-dog and the cat, accompanied by nervo-muscular symptoms or gradual inanition, with or without any apparent specific lesion on postmortem. A true solution of the details of such intoxication is only possible after we have a more thorough knowledge of the chemico-physiologic changes in the body. It is, however, quite appreciable that there are some conditions which favor the production of *ptomaïns* in the intestinal tract, from food fermentation, which may be poisonous in the same manner in which the toxin of the tetanus bacillus, remaining localized on the surface, is absorbed and intoxicates the nervous system.

The *leucomaïns*, the toxic compounds formed within the body, are an important factor. The ingredients of the food taken into the alimentary canal undergo chemic changes when digested: they are absorbed; the cells of the different tissues and organs select those materials necessary for their sustenance, and change these materials into their own proper cell-constituents. When the cell-tissue or organ acts or is exercised there are still other chemic changes: the nutritive pabulum a moment before absorbed is now destroyed, oxidized, or converted into poisonous compounds which, when not promptly eliminated from the system and allowed to accumulate (uræmic poisoning), or not neutralized by other products, produce certain symptoms. Again, the surplus albumin, fat, sugar, etc., not needed to maintain the nutritive balance, must be converted into other compounds for elimination from the

[1] Read before the semi-annual meeting of the Pennsylvania State Veterinary Medical Association, Pittsburg, September, 1898.

system. In this vast laboratory of chemistry in the animal body, if the function of an organ be increased, diminished, or perverted, any one of such poisonous products may be formed in excess of its normal quantity or checked in its elimination or new compounds may be formed. A disturbance of the function of one organ, although of little import in itself, may alter the function of some other organ. We have abundant illustrations of the "functional sympathy" among all the organs and the altered functions resulting from its disturbance.

In order to show the complexity of the process of the organic disassimilation, as well as the many chemical compounds, the albuminous matter of the food is decomposed into:

1. Nitrogen—containing acids—uric, hippuric, and oxaluric; the last, by losing its nitrogen, becomes oxalic acid.

2. Sugar, whence the formation of lactic, stearic, capric, valeric, succinic, oxalic, and carbonic acids, or the amides (starches) of these acids—leucin, tyrosin, glycocoll.

3. Cholesterin, whence the volatile fatty acids—caprilic, capric, valeric, butyric, propionic, acetic, and oxalic.

4. Sulphur, whence sulphates by oxidation.

The fatty matter is decomposed into glycerin; the latter forms water, carbonic acid, and volatile fatty acids—oleic, stearic, and palmitic.

Starch forms dextrin and glucose, and, finally, lactic, butyric, acetic, and oxalic acids.

The *leucomaïns*, or toxins elaborated in the animal tissues during the process of selection by the cells, from the circulating fluid of those materials which they need, and of transforming the surplus, as well as the waste materials formed in these processes, into compounds capable of elimination from the system, are to the animal body what the toxins are to microbes, and the alkaloids, such as aconite, pilocarpine, to the higher plants.

The principal leucomaïns found in the body are: 1. Adenin; 2. Plasmain; 3. Sarcin, found in the white cells of the blood, the liver, spleen, thymus, kidney, and heart; also in growing vegetable shoots. 4. Xanthin. 5. Pseudoxanthin. 6. Paraxanthin, isomeric with theobromin. 7. Hetroxanthin, found in small quantities in the urine of the dog. 8. Guanin. 9. Carnin, found in yeast and extract of meat. 10. Creatin in the muscles, brain, blood, and at times in the urine. It has no toxic properties. 11. Creatinin, in the muscles, blood, organs, urine, and some animal secretions. It is a strong base and, when excessively alkaline, possesses toxic properties (Cadeac).

Various other principles, endowed with great toxic properties, but which have not been isolated, exist in the bile, urine, etc. In a culture of bacteria the latter will cease to vegetate when the medium becomes saturated with toxin secreted above a certain point. Likewise, the living animal tissues, when they become saturated above a certain point with one or more of the toxic principles elaborated within the body or absorbed from the intestinal tract, show perversion of function and symptoms of disease. More than this, these toxins are capable of producing anatomic alterations in the organs, as is seen in the liver and kidneys. Venin, the animal toxin of certain snakes, for example, produces nephritis in susceptible animals.

These various chemic processes in the body are under the control of definite functions and properties possessed by the cells. When such properties and functions become perverted, either by an altered supply of nutritive material, deviation in the intrinsic functions of the protoplasm, or in the excretory functions, the nature and regularity of the phenomena of nutrition become altered and the body becomes saturated with obnoxious materials. The organs most essential to nutrition, the emunctories and nervous system, are probably those frequently at fault primarily. Again, some rich food-stuffs are converted into toxins formed either in the digestive tract or after absorption into the system. Excessive absorption of nutrients from the intestinal mucous membrane cause plethora of the liver, prevents the subsequent assimilation and disassimilation, and disturbs the glandular secretions. Wheat and barley fed in excessive quantity produce laminitis; vetches are said to cause roaring. Meat, wholesome at the time of ingestion, may, when it remains too long in the stomach or when the digestive secretions are altered or insufficient, become poisonous. Whatever be the source of the toxic agent which circulates through the system, the consecutive phenomena vary according to its nature and the organs affected by it.

The organs offer a different susceptibility to the diverse toxic products. The phenomena may be due to a local or to a reflex action. For example, stimulation or neuritis of the central end of the several pneumogastric or the first dorsal nerve is followed by sugar in the urine; stimulation of the fourth ventricle, by sugar and albumin in the urine. To give many specific illustrations of autointoxication and prescribe therapeutic means require more knowledge of the physiologic processes than we now possess. A few may be attempted. For instance, *nervine*, resulting from the decomposition of lecithin, a nerve-tissue by-product, is exceedingly toxic, one-sixth of a grain destroying a cat. Cholin, found in the blood, muscles, and bile of the pig, is also virulent. Other illustrations of toxic formations in the system, to which they are detrimental unless their effects are neutralized, are the thyroid and pituitary bodies, suprarenal capsules, pancreas, spleen, and bone-marrow. Numerous experiments have shown that in some animals—dog, cat, rabbit, and some herbivora—the thyroid body contains toxic principles which are capable of producing goitre, myxœdema, cachexia, and, in young animals, arrest of development; that if the thyroid be removed, these principles elaborated in the organs will accumulate in the system with the above characteristic symptoms. One of the products isolated from the thyroid body is thyreoproteid. It is the product of organic changes in the body, and, when the thyroid is removed, produces an acute intoxication. Another substance isolated, but formed in the thyroid, is thyroidine; it contains nitrogen and iodin. It acts upon the thyreoproteid as a ferment, which it thus renders innocuous or neutralizes like a sort of antitoxin. Thus, when the thyro-antitoxic function becomes disturbed, the principles ordinarily neutralized by the products of the thyroid accumulate in the system, and *intoxication* is the result. We can offer no better explanation than this for such conditions as cholæmia (jaundice), gout, rheumatism, stercoræmia (fecal intoxication), diabetic coma, uræmia, and probably also chorea, neurasthenia, asthma, and idiopathic anæmia, although the toxic nature of the substance has not in all cases been determined. We find an illustration of this in azoturia. If Möller's theory of the excessive acidity of blood is correct, it is an intoxication.

Then we have the various diatheses, the arthritic, herpetic, and scrofulous. The nutritive processes are disturbed, the system is overloaded with materials which the cells cannot utilize, and rheumatism, diabetes and obesity, and deposits of inorganic salts result. In the herpetic diathesis we have the various skin troubles, eczema, etc. Herpetic diseases were described as caused by an excess of lactic and uric acids, etc., and by our sister practitioners are treated principally with alkalies. In aged dogs the nutritive chemic functions are not performed with the same activity and accuracy as in the young. In dogs and often in kittens we have obesity and eczema. During the hot season, when the cutaneous circulation is active and loaded with such materials, we meet skin troubles; in the cold season, when the pulmonary circulation is increased at the expense of that of the skin, we find catarrh of the bronchial mucous membrane, which is seen to appear when the skin trouble disappears. We can understand why local treatment is not permanent. In young dogs we find ulceration of the cornea. In the horse we have acute and chronic affections of the skin, such as grease and urticaria, and, perhaps, lymphangitis. When a horse is "out of condition," a polite term to cover our ignorance, may there not, owing to excess or a change of food or other adverse influences upon the system, be a disturbance in the nutritive forces of the tissue, the production of a milk poison, which checks the digestive and cutaneous secretions, depress the circulation and the nervous system, and, hence, the loss of appetite, dry coat, slow circulation, and general depression. We see cases, especially in the horse, also in other animals, accompanied for a week or more with absolute loss of appetite, little or no fever, extreme depression, emaciation, probably a yellowish coloration of the mucous membranes, and an acute intoxication of many of the tissues; also low, adynamic fevers sometimes lasting for some time.

Evidence of autointoxication appears most frequently in the carnivorous house pets. This is especially facilitated by their unnatural condition of living, lazy habits, and errors of diet. Frequent indigestion and gastro-intestinal catarrh, constipation, fetid diarrhœa indicate altered nutritive function and the frequent probability of the absorption of or the formation within the body of toxic products. These most frequently assume the nervous and the cachectic forms. In middle-aged and old dogs I have most frequently found convulsive symptoms to dominate. They are not the benign convulsions seen in young animals. The animal may be attacked suddenly by spells of exciting, running around the house, followed by frequent paroxysms of convulsions, muscular relaxation, and death. Again, I have seen allied conditions complicating other diseases. For example, the patient may be convalescent from broncho-pneumonia, progress favorably, but at a given time and without any aggravations of the primary disease, which may have disappeared, the constitutional symptoms relapse with gradual loss of appetite, weakness, emaciation, and death.

In cats these phenomena may assume the nervous or the cachectic form. In the former, the pet appears frightened, with slight convulsions, inappentency, and death in the course of some days. In the latter, without any apparent cause, the animal refuses to eat shows emaciation, shedding of fur, constipation, or diarrhœa, cachexia for weeks before death; also catarrhal condition of the mouth and pharynx, with discharge from the nose. Temperature toward the termination of the disease subnormal. In a few instances I have been called to treat the animal for severe eczema, soon after the treatment was commenced, and no drugs were given that could explain the symptoms convulsions, and death in about a week followed. Were the two conditions due to the same toxic agents? Again, stercoræmia, infection from the intestines, may in these animals cause primary troubles or complicate recovery from other diseases.

I have made a few postmortems of a number of such animals and found the usual lesions that toxic infections present. The liver and kidney were the seat of lesions. The former in one cat was enlarged, fatty (infiltration), the proper tissue having almost disappeared. The kidneys in both dogs and cats were altered; the entire cortex, the secreting portion (Malpighian bodies and convoluted tubules) were fatty, and the line of separation from the medullary portion was very abrupt. We have in the kidney evident lesions, nephritis, and fatty degeneration, showing that some irritating toxic agent has been continually excreted by these organs, and when the latter were no more able to remove this principle from the blood, death soon followed. We see analogous lesions in animals after the prolonged administration of mineral poisons, such as arsenic and mercury. In other cases no decided alterations may be found. Such symptoms and lesions in cats I have seen very frequently.

The treatment is, perhaps, contingent. If such alterations as I have described have progressed far, it is of little value. Digestive and systemic tonics and cardiac stimulants are given. Bowel antisepsis with thymol, salol, creolin, etc., are indispensable; also diuretics and alkalies, to neutralize any toxic principle and assist in its excretion; regulation of the bowels and stimulating baths.

It is not my intention to classify promiscuously too many conditions under the head of autoinfection, but that it is an important factor in pathogenesis and may include many conditions heretofore not understood.

